# Helping families thrive while developing a strong working alliance: The benefits of Recipe 4 Success

**DOI:** 10.1002/imhj.70105

**Published:** 2026-06-08

**Authors:** Sarah M. Braaten, Robert L. Nix, Lori A. Francis, Mark E. Feinberg, Michelle L. Hostetler, Cynthia A. Stifter, Sukhdeep Gill

**Affiliations:** ^1^ University of Wisconsin‐Madison Madison Wisconsin USA; ^2^ Pennsylvania State University University Park Pennsylvania USA; ^3^ Penn State York York Pennsylvania USA

**Keywords:** working alliance, curriculum, home visiting, early head start, interventions, poverty

## Abstract

This study explored whether the strength of parent‐home visitor working alliances might alter the benefits of Recipe 4 Success, a highly structured food‐based curriculum designed to promote parents’ sensitive scaffolding, responsive food parenting practices, toddlers’ self‐regulation, and healthy eating habits. This study included 242 parents and their toddlers residing in the United States, most of whom were living in poverty (37% white, 25% Black, 19% Latiné, 17% Multiracial, and 2% Asian; median income = $1555 per month). Families were randomly assigned to Recipe 4 Success or usual practice home visits within home visitor caseload. Home visitors tended to rate their working alliance as very strong versus less strong. Within‐group regression equations revealed that, among families with a less strong working alliance, Recipe 4 Success was more effective than usual practice home visits in improving parents’ sensitive scaffolding, responsive food parenting practices, and toddlers’ self‐regulation. In contrast, among families who had a strong working alliance, Recipe 4 Success was more effective than usual practice home visits in changing toddlers’ healthy eating habits, including reducing body mass index for toddlers with overweight/obesity. These findings highlight how families with varying strengths of working alliances may benefit differently from highly structured evidence‐based curricula.

## 1 INTRODUCTION

This study explored whether the strength of the parent‐home visitor working alliance might alter the effectiveness of Recipe 4 Success, a preventive intervention embedded within home visiting programs. A strong working alliance describes the ideal relationship between a human service professional, such as a home visitor, and a client, such as a parent, in which there is trust, mutual respect, and agreement on goals (Bordin, 1979; Roggman et al., 2016). Within two‐generation home visiting programs for families living in poverty, a strong working alliance is considered the cornerstone of collaboration and a driving force of family progress. As home visitors establish trust and articulate shared goals, parents are more receptive to suggestions from their home visitor and are more likely to engage in responsive parenting behaviors and positive parent‐child interactions (Korfmacher et al., 2007). Without a strong working alliance, parents tend to be less satisfied with their home visits, unengaged, and are more likely to drop out (de Greef et al., 2018).

It can be challenging for home visitors to establish a strong working alliance—particularly building and maintaining trust and mutual respect—with parents who have had negative and possibly traumatic experiences in the past with human service professionals, authority figures, family members, or romantic partners (Jack et al., 2002; Shanti, 2020). It may be difficult to establish a strong working alliance when the goals of home visits are unclear, misunderstood, or do not meet a family's self‐perceived needs (Jack et al., 2002). Sometimes, it also can be more challenging for home visitors to establish a strong working alliance with parents from different socioeconomic, racial, ethnic, or cultural backgrounds (Cook et al., 2023; Shanti, 2020). To cultivate a strong working alliance, home visitors strive to be dependable, professional, and family‐focused, and they encourage the parent to take on a partner role, in which home visitors acknowledge the parent as the undisputed expert with unique insight into their child's and family's needs and circumstances (Jack et al., 2002). To support home visitors’ efforts in cultivating a strong working alliance, home visiting programs often offer ongoing reflective supervision (Tomlin et al., 2016) and considerable professional development training (Ingoldsby, 2010).

In addition to nurturing a strong working alliance with each family in their caseload, home visitors are tasked with delivering evidence‐based curricula (U.S. Department of Health and Human Services [https://homvee.acf.gov/models/]). The curricula vary in terms of how structured they are and how much sanctioned flexibility they allow in tailoring lessons. However, all curricula are developed by master practitioners and represent some of the latest empirical research on early childhood development, adult learning principles, and tried‐and‐true parent‐child interaction activities. Curricula often incorporate active parent coaching strategies, which are some of the most effective means of promoting positive behavior change (Walsh et al., 2022). Moreover, as the name implies, evidence‐based curricula have been rigorously evaluated for feasibility and effectiveness: They have been shown to produce actual change among hundreds or thousands of prior families with similar characteristics facing similar circumstances.

**Key points**

- Recipe 4 Success improved several aspects of parents’ sensitive scaffolding behaviors, responsive food parenting practices, and toddlers’ self‐regulation among families with a less strong working alliance.
- Recipe 4 Success improved toddlers’ healthy eating habits, including a reduction in body mass index, among families with a strong working alliance.

**Statement of Relevance**

This study is highly relevant to the field of infant and early childhood mental health because it provides novel insights about how a preventive intervention, embedded within an existing early childhood home visiting model, can provide unique support to families with a less strong parent‐home visitor working alliance, as well as families with a strong working alliance. This research contributes to the field by encouraging the use of structured curricula to support high‐quality home visiting services.

Unfortunately, however, home visitors sometimes struggle to balance nurturing the working alliance with delivering a standardized curriculum (Barak et al., 2014). There are at least four ways in which this tension plays out. First, when home visitors are just starting to work with a family or are still defining family goals, they may prioritize getting to know and truly understand the parent and child by spending time together more informally, rather than focusing on specific curriculum content (Barak et al., 2014). Second, during that time home visitors are finding ways to connect with a family, they might struggle to identify what is going to be most helpful among the curriculum content they do put together and deliver. Third, when home visitors are unsure of their relationship with a particular family, they may be overly cautious in what they ask of parents, such as by choosing to model rather than actively coach specific child‐focused interactions (Braaten, 2024). Fourth, and relatedly, when the home visitor does not believe a curriculum is meeting specific family needs or preferences, they may be tempted to adapt lessons in other idiosyncratic ways (Barak et al., 2014). This tension between attending to the working alliance and delivering a standardized curriculum can sometimes prompt home visitors to deviate from curriculum guidelines in ways that ultimately undermine home visit effectiveness and do not serve families well (Bierman et al., 2006). There is considerable empirical evidence that has accumulated over decades on the limitations and inherent biases of professional or clinical judgment about what a family might need and how to tailor lessons to meet those needs (Grove, 2005).

### 1.1 The Recipe 4 Success preventive intervention

Recipe 4 Success was created through a community‐based participatory research collaboration involving families enrolled in home visiting, home visitors, program administrators and university researchers (Nix, 2020). It was designed to help home visitors be more effective with all of the families they worked with, regardless of the strength of their working alliance, especially around the issues of healthy eating habits and child obesity. It often was challenging for home visitors to talk with parents about sensitive topics such as what they were feeding their children, and the resources that home visitors had available did not resonate with families or were not appropriate for families living in poverty.

In developing Recipe 4 Success, it was emphasized repeatedly that the relationship home visitors had formed with the families they worked with was sacrosanct. A new intervention could not do anything that might jeopardize the working alliance home visitors had already formed or were working to build.

Early on, we determined that a new intervention that merely provided information about responsive food parenting practices and healthy eating habits might come across as too preachy or judgmental and was unlikely to change behavior. We believed an intervention was more likely to be useful if it was broader in its focus and also targeted factors related to healthy eating habits. As a result, we created a preventive intervention that simultaneously sought to enhance the development of parents’ sensitive scaffolding, responsive food parenting, and toddlers’ self‐regulation, as sensitive scaffolding supports the development of self‐regulation (Mermelshtine, 2017), and responsive food parenting and early self‐regulation predict healthy eating habits over time (Francis & Susman, 2009; Hurley et al., 2011). We learned from early conversations that parents typically signed up for home visiting programs because they wanted their children to be well‐behaved and to do well in school. Because self‐regulation predicts physical health, academic achievement, and social adjustment (Robson et al., 2020), this broader focus, with an emphasis on self‐regulation as a unifying construct of many developmental processes during the toddler period, would make the intervention appealing to all families, regardless of their specific interest in promoting healthy eating habits.

We decided to organize Recipe 4 Success around carefully structured and sequenced food preparation activities for several reasons. First, activities like carefully measuring ingredients and stirring them together without spilling would provide multiple opportunities for parents to practice sensitive scaffolding of their toddlers’ self‐regulation and learning. At the same time, the activities would provide toddlers with a variety of opportunities to practice skills underpinning self‐regulation, like paying attention, controlling their bodies, and cooperating with others. Second, we believed that parents would be more actively involved in food preparation activities than usual practice home visits because the food preparation activities would typically occur in their kitchen, where only they knew where everything was, and it would be harder for home visitors to take the lead. Third, toddlers would be exposed to multiple new fruits and vegetables. Fourth, toddlers are more likely to eat new foods they help prepare (Knai et al., 2006). And, finally, we could tap into the ways that preparing and eating food together comprise a primal source of nurturance, joy, and celebration across cultures.

In focus groups, we observed considerable heterogeneity in the ways that parents interacted with their toddlers (Nix, 2020). For example, some parents were very quiet and reserved in their interactions with their toddlers, and other parents were highly engaged, but sometimes commanding and overly directive. We decided to teach four specific skills adapted from parent‐child interaction therapy because they were so effective for all parents and reinforcing for all toddlers (McNeil & Hembree‐Kigin, 2010). We hoped that, if parents could master these skills, they would be perceived as more sensitive and responsive by their toddlers and more effective in scaffolding. These four skills were describing everything the toddler was doing to demonstrate the parent is paying attention and to encourage the toddler to persist with challenging tasks; reflecting and expanding on everything the toddler tried to communicate to facilitate language use and problem solving; providing specific praise for effort to promote a growth mindset; and offering concrete choices to enhance toddlers’ autonomy and investment in the activities. At the beginning of the intervention, we had home visitors teach and practice these four skills, and throughout subsequent lessons, we provided sample scripts that identified the many times home visitors could prompt parents to continue using the four skills.

In those ways, we hoped Recipe 4 Success would help home visitors nurture strong working alliances with families and make it easier to deliver a standardized curriculum. In particular, the intervention focused on topics, like self‐regulation and healthy eating habits, relevant to all families. We provided home visitors with specific language they could use with families to ensure goal alignment. Our food preparation activities prompted parent engagement and were less amenable to major tailoring. Insights from adult psychotherapy suggest a strong working alliance is enhanced, not undermined, by doing the kind of evidence‐based therapeutic work those results in substantive change (Feeley et al., 1999). Clients tend to feel closer to their therapists or counselors once they notice a reduction in symptoms and enhancement in well‐being. We hoped this would be the case in home visiting as well, with parents feeling more connected to their home visitor once they experience positive changes in themselves and their toddlers. We were encouraged when, in two separate randomized controlled trials, Recipe 4 Success, compared to usual practice home visits, had a more positive impact on parents’ sensitive scaffolding, responsive food parenting practices, toddlers’ self‐regulation, and healthy eating habits (Nix et al., 2021, 2024).

### 1.2 The present study

The present study had three aims. First, we wanted to examine the nature of the parent‐home visitor working alliance. Based on how important home visitors believe a strong working alliance is, how much effort they put into nurturing a strong working alliance, and the fact that home visitors were hired because of their abilities to build trust in a relationship, we hypothesized there would be a very strong working alliance for most families. We did not expect much variability in the range of working alliances, as families would be unwilling to engage in something as intimate and vulnerable as home visits if a high baseline of mutual respect and goal alignment was not present. Second, we wanted to determine what family characteristics were related to the strength of the working alliance. We hypothesized that some risk factors, such as parent depression, might affect the working alliance, as similar risk factors have in previous studies. However, we also knew that home visitors are professionals capable of connecting with all families in their caseload, so we did not expect too many characteristics to be related to working alliance. Third, this study explored whether the strength of the working alliance might alter the overall pattern of positive intervention effects in Recipe 4 Success. We hypothesized that families with a less strong working alliance might benefit from participating in Recipe 4 Success because the tensions that often exist between nurturing a working alliance and delivering a standardized curriculum should be less problematic because we designed the curriculum so it would be relevant to and easy to deliver to all families.

## 2 METHOD

This study used data from the second clinical trial of Recipe 4 Success (Nix et al., 2024). The clinical trial was approved by the Institutional Review Boards of [masked for review] and pre‐registered at clinicaltrials.gov, [masked for review].

### 2.1 Participants

This study included 242 parents and their toddlers. Thirty‐seven percent of families were non‐Hispanic white; 25% were Black; 19% were Latiné; 17% were Multiracial; 2% were Asian American; and less than 1% was Native American. Ninety‐one percent of parents were biological mothers; 9% were fathers or other relatives. Fifty‐five percent of parents were married or lived with a partner. Thirty‐five percent of parents had any education or advanced training beyond high school. Sixty percent of parents did not work outside the home; 20% had a part‐time job; and 20% had a full‐time job. Most families lived below the federal poverty level with a median household income of $1555 per month and a median income‐to‐needs ratio of .75.

Fifty‐one percent of toddlers were girls. On average, children were 31 months old (*SD* = 3.76, range = 21–39) at the start of the study.

### 2.2 Recruitment and condition assignment procedures

All families in this study were enrolled in Early Head Start home visits in seven cities, small towns, or rural areas of Wisconsin or Pennsylvania. Families were matched with their home visitors upon enrollment in Early Head Start, based on factors such as caseload availability, home visitors’ particular skills and experience, and shared language, race, and ethnicity. It was those home visitors who initially approached eligible families with a toddler about the possibility of participating in this study. If families were interested, research project staff members met with them to provide additional information and obtain consent.

Families were randomly assigned to study condition within home visitor caseload, based on a coin toss conducted by the project manager. This approach would balance any attributes or variability among home visitors that might affect either the establishment of a strong working alliance or the delivery of an evidence‐based curriculum. Families were assigned to participate in Recipe 4 Success during their regular home visits as delivered by their home visitor or to continue receiving usual practice home visits, also delivered by their home visitor. In almost all cases, those usual practice home visits followed the evidence‐based Parents as Teachers curriculum and focused on facilitating positive parent‐child interactions to enhance children's physical, cognitive, language, and social‐emotional development (Wagner & Clayton, 1999). Families in Recipe 4 Success did not receive extra home visits or extra‐long home visits. Rather, Recipe 4 Success covered similar content as usual practice home visits, but in a different format.

### 2.3 Intervention procedures

Recipe 4 Success consisted of 12 highly structured, carefully sequenced, and scripted food preparation lessons, which lasted about 30–45 min of a typical 90 min home visit. We provided all materials, including food ingredients, needed for each lesson.

Prior to delivering Recipe 4 Success, home visitors participated in two‐day training. Throughout the intervention, home visitors engaged in weekly conference calls with the principal investigator and continued to receive individual and group supervision as part of Early Head Start.

To assess fidelity of implementation, we had home visitors record three home visits, corresponding to Lessons 4, 7, and 10 of Recipe 4 Success. We then had lab‐based research assistants rate those audio recordings, with double coding of 15% of all recordings. Research assistants noted the presence or absence of 15 essential components of Recipe 4 Success, such as reviewing practice from the previous week, discussing each of the two different aspects of self‐regulation or healthy eating habits, prompting the parent to use each of the four cores parenting skills, and making an action plan for the following week. Across the lessons, home visitors completed an average of 75% of the curriculum's essential components (intraclass correlation coefficient [ICC] = .96).

### 2.4 Assessment procedures

All assessments were conducted in families’ homes by a diverse team of project interviewers who were selected based on their experience working with families and their interpersonal and organizational skills. Project interviewers completed extensive training before conducting any assessments on their own, and they participated in ongoing supervision. They were masked to study condition.

Families completed baseline and post‐intervention assessments, occurring at least one week before and one week after the 12‐week curriculum. The assessments included a parent interview, direct testing of toddlers’ self‐regulation, structured parent‐child interaction tasks, and a shared unfamiliar snack. During the assessments, home visitors also completed standardized ratings about the families they served. At the end of each assessment, project interviewers completed ratings about what they observed while in the homes. Over the following week, they completed two follow‐up telephone calls.

### 2.5 Measures

This study included one measure of home visitors’ perception of their working alliance with parents, assessed at baseline, and several measures of family characteristics we thought might be related to working alliance, also assessed at baseline. Because our goal was to determine whether the strength of that working alliance might alter intervention effectiveness, this study also included all 12 of the validated parent and toddler outcomes from the most recent clinical trial of Recipe 4 Success (Nix et al., 2024), assessed at both baseline and post‐intervention.

#### 2.5.1 Home visitors’ perception of the working alliance

Home visitors’ perception of their working alliance with parents was assessed with an adapted version of the Working Alliance Inventory, Short Form (Tracey & Kokotovic, 1989), which included 11 items, such as “This parent and I agree on what is important for them to work on,” rated on a 4‐point Likert scale with 1 = *rarely*–4 = *almost all the time* (*α* = .93).

#### 2.5.2 Family characteristics potentially related to working alliance

This study included several family characteristics that were potentially related to the strength of the parent‐home visitor working alliance. Some of the family characteristics, all based on parent reports, were family race and ethnicity (with families that included more than one race being considered multiracial); whether the parent was single versus living with a spouse or romantic partner; family size; whether the parent had any post high school education or training; whether the parent worked outside the home at a full‐ or part‐time job; number of months in the previous year the family had difficulty paying for food, rent, or utilities; income‐to‐needs ratio; toddler gender; and toddler age.

Other family characteristics were parent depression, parenting stress, household chaos, all based on parent reports, and the need for therapeutic services, based on ratings by project interviewers. Parent depression was assessed with the Center for Epidemiological Studies‐Depression scale (Radloff, 1977), in which parents rated 20 items, such as “During the last week, how often did you feel depressed?” with 0 = *rarely (<* *1 day)*–3 = *almost all of the time (5–7 days*; *α* = .90). A total score of 16 or more on this measure indicated clinically significant symptoms of depression. Parenting stress was assessed with an adapted version of the Daily Hassles scale (Crnic & Greenberg, 1990), in which parents rated five items, such as “Children get in the way or interfere with chores,” with 1 = *rarely*–4 = *almost all the time* (*α* = .66). Household chaos was assessed with an adapted version of the Confusion, Hubbub, and Order scale (Matheny et al., 1995), in which parents rated six items, such as “You can't hear yourself think in your home,” with 1 = *rarely* and 4 = *almost all the time* (*α* = .77). The need for therapeutic services or case management was assessed with an adapted version of the Post‐Visit Inventory (Dodge et al., 1990), in which our research‐team project interviewers relied on what they had observed during their time in families’ homes to rate three items, “The parent could benefit from parent training,” “The parent might need social work intervention (i.e., help with housing),” and “The parent might need mental health services (i.e., therapy for depression or couples counseling),” with 1 = *not at all*–10 = *very much* (*α* = .80).

#### 2.5.3 Parent and toddler outcomes

The first three of the 12 outcome measures in this study represented different aspects of parents’ sensitive scaffolding, which were assessed via three 3 min video recorded parent‐child interaction tasks (e.g., bowling with stuffed pins and a ball, building a tower with different‐sized blocks, and completing a shape sorter puzzle). Each task was coded by two independent research assistants and reliability was computed for each item. Final scores for each measure were computed as an average of the individual items across the three parent‐child interaction tasks and the two research assistants. (1) The first measure of sensitive scaffolding was use of the **core parenting skills** hypothesized to support children's self‐regulation. It was assessed with four items, such as “Provided specific praise for effort,” which were adapted from similar items used to assess the quality of parent engagement in parent‐child interaction therapy (McNeil & Hembree‐Kigin, 2010). Items were rated on a 3‐point Likert scale with 0 = *none*, 1 = *once*, and 2 = *twice or more* (ICC = .72– .87; *α* = .76). (2) The second measure, parents’ **learning support** behaviors, was assessed with seven items, such as “Offers suggestions or ideas,” which were adapted from a similar measure of observed learning support (Bierman et al., 2015), and rated on a 5‐point Likert scale with 1 = *almost never*–5 = *almost always* (ICC = .62– .91; *α* = .89). (3) The third measure, parents’ **technical scaffolding,** reflected how well parents structured the interaction task to facilitate their toddlers’ success and independence and was assessed with one global rating from a scale of scaffolding behaviors (Hoffman et al., 2006), with response options ranging from 1 = *parent exhibits characteristic ineffectiveness*–5 = *parent exhibits characteristic effectiveness and meets the toddler's needs almost the entire time* (ICC = .76– .79).

The fourth and fifth outcome measures represented food parenting practices and were assessed during a video recorded shared snack task in which parents introduced two unfamiliar healthy foods, such as dried seaweed or prunes, to their toddler. (4) Parents’ **responsive/non‐coercive strategies** represented a tally of 10 behaviors, such as “Modeling the enjoyment of the new food,” which were derived from a review of factors associated with toddlers’ interest in new foods and negatively related to child obesity (Hurley et al., 2011). These items were rated as 0 = *absent* and 1 = *present* (ICC = .73– .88). (5) The other measure of food parenting practices assessed **overall sensitivity**, which reflected parental warmth, acceptance, and willingness to follow the child's lead regarding what they did or did not want to eat, and was created for the first clinical trial of Recipe 4 Success (Nix et al., 2021), with response options ranging from 1 = *not at all*–5 = *very much* (ICC = .84).

The sixth through ninth outcome measures represented different aspects of toddlers’ self‐regulation. (6) Toddlers’ ability to **delay gratification** was assessed with the validated snack delay task (Kochanska et al., 2000), in which project interviewers placed a single M&M in front of the child and told them they could eat it but needed to wait until time was up, with trials of 5, 15, 30, and 60 s, each scored as 0 = *ate M&M before time was up* or 1 = *waited full time* (*α* = .87). (7) **Task orientation** was assessed with the task orientation/regulation subscale of the Infant Behavior Record (Bayley, 1969; Stifter & Corey, 2001) and was based on a rating of overall interest and a rating of overall attention by project interviewers, completed at the end of the entire assessment battery. Response options for interest ranged from 1 = *does not indicate interest in objects*–9 = *reluctantly relinquishes test materials*, and response options for attention ranged from 1 = *fleeting attention*–9 = *long continued absorption (r *= .59*, p < .001)*. (8) **Compliance** was assessed with the compliance subscale of the Infant‐Toddler Social and Emotional Assessment (Carter et al., 2003), in which parents rated eight items, such as “Obeys when asked to stop being aggressive,” with 1 = *not true/rarely true*–3 = *very true/often* (*α* = .66). (9) Toddlers’ **dysregulation** was assessed with the dysregulation subscale of the Infant‐Toddler Social and Emotional Assessment (Carter et al., 2003), in which parents rated 27 items, such as “Hard to soothe when upset,” with 1 = *not true/rarely true*–3 = *very true/often* (*α* = .88).

The final three outcome measures represented different aspects of toddler's healthy eating habits, which are highly related to self‐regulation. Two of the three measures were based on three 24 h dietary recalls, one in person and two over the telephone, in which project interviewers asked parents about what their toddler had done throughout the day, including what they had eaten and drank. (10) One measure of healthy eating habits, created for the first clinical trial of Recipe 4 Success (Nix et al., 2021), represented an average of the **percentage of meals and snacks consumed across the three days that included a fruit, a vegetable, and a source of protein**. (11) Another measure of healthy eating habits, created for the second clinical trial of Recipe 4 Success (Nix et al., 2024), represented the **percentage of days in which toddlers helped prepare their meals or snacks**. (12) The final measure assessed toddlers’ **body mass index (BMI)** based on the Centers for Disease Control and Prevention BMI‐for‐age percentiles. For this outcome, analyses were only conducted with the subsample of toddlers with overweight or obesity at baseline (i.e., BMI at or above the 85^th^ percentile [*n* = 103]), for whom a reduction in BMI might be desirable.

### 2.6 Plan for analysis

In the first stage of data analyses, we used descriptive statistics to examine the distribution of working alliance in the sample and whether there was support for our hypothesis that most families would fall into two groups: as having either a strong, nearly optimal working alliance or as having a slightly less strong, but still good working alliance.

Second, we used independent two‐sample *t*‐tests (for continuously distributed measures) and chi‐square tests (for dichotomous measures) to examine how families with a strong versus less strong working alliance differed on key family characteristics.

Third, we estimated multivariable regression equations for the 12 parent and toddler outcomes included in the second clinical trial of Recipe 4 Success (Nix et al., 2024). The independent variables in each regression equation were the baseline level of the outcome and intervention status, with 1 = *Recipe 4 Success* and 0 = *usual practice home visits*. To best highlight distinct patterns of findings, we estimated regression equations for families with a less strong working alliance. Then, we re‐estimated regression equations for families with a strong working alliance. All variables were standardized within working alliance subgroups, *M* = .00, *SD* = 1.00, so that each beta, *β*, would be comparable to a Cohen's *d* intervention effect size, representing the difference between families in the intervention and control groups as a proportion of a pooled standard deviation, controlling for the baseline level of the outcome. In the clinical trial of Recipe 4 Success, outcomes were pre‐registered, and nine of the 12 outcomes had a probability value below .05, and the other three outcomes had probability values between .05 and .10. Given that clear pattern of results in the clinical trial and our goal to determine whether differences in working alliance might alter those intervention effects, we did not adjust probability values in this study to account for the additional models we estimated. Doing so could have made Recipe 4 Success appear ineffective for all families. At the same time, we were careful to only highlight and interpret individual intervention effects that were part of a clear pattern and clinically meaningful.

To formally test whether working alliance altered Recipe 4 Success intervention effects, we again re‐estimated the regression equations but included all families and added two new independent variables: a dichotomous indicator of working alliance, with 1 = *strong working alliance* and 0 = *less strong working alliance*, as well as an interaction term, representing the product of intervention status and that dichotomous indicator of working alliance. The probability value associated with the interaction term would indicate whether families with a strong and a less strong working alliance produced intervention effects that were statistically significantly different from one another.

Sensitivity analyses were conducted to determine whether results differed once we accounted for the fact that families were nested within home visitor caseloads. However, Level 2 variance was often so small it could not be estimated. When it could be estimated, the variance hardly changed parameter estimates of intervention effects. Likewise, sensitivity analyses were conducted to determine whether results differed once we controlled for multiple family‐level covariates, such as race and ethnicity, marital status, parent education, and parent employment, family financial strain, parents’ depression, and household characteristics. No covariates were consistent statistically significant predictors of the parent and toddler outcomes, and the inclusion of those covariates did not affect the pattern of results.

## 3 RESULTS

This study included data on home visitors’ perception of working alliance for 241 of 242 (99%) participating families and data on parent and toddler post‐intervention outcomes for 229 of the 242 (95%) families. As hypothesized, home visitor ratings on the Working Alliance Inventory tended to be quite high, *M* = 3.47, *SD* = .47, on a 4‐point Likert scale, with the mean and median scores being the same. Visual inspection of ratings on the Working Alliance Inventory indicated a clear bimodal distribution, with many families clustering just above the mid‐point of the scale and all other families receiving near optimal ratings. One hundred and ten families were below the median and were considered to have a less strong working alliance, *M* = 3.02, *SD* = .29, with the behavioral anchor of 3 = *often*. One hundred thirty‐one families were above the median and were considered to have a strong working alliance, *M* = 3.84, *SD* = .17, with the behavioral anchor of 4 = *almost all the time*. The means of the two subgroups differed by 1.74 standard deviations ([3.84–3.02] / . 47 = 1.74), and the variance within each subgroup was small, indicating substantial differentiation among the two subgroups of families.

Independent two‐sample t‐tests and chi‐square tests, presented in Table 1, revealed the strength of the parent‐home visitor working alliance was related to several family characteristics. Latiné families tended to have a strong parent‐home visitor working alliance rather than a less strong working alliance, 70% versus 30%, respectively, *p* (of difference) = .02. Parents with any post‐high school education tended to have a strong, compared to less strong, working alliance, 64% versus 36%, respectively, *p* = .03. In addition, families with older toddlers tended to have a strong, compared to less strong, working alliance, *M* toddler age = 31.80 months versus 30.12 months, respectively, *p* = .001.

**TABLE 1:** Family characteristics related to strength of working alliance.

	Less strong working alliance	Strong working alliance	*P*‐value of difference
Family race and ethnicity			
Non‐Hispanic White	54%	46%	.03
Black	42%	58%	.56
Latiné	30%	70%	.02
Multiracial	50%	50%	.54
Asian	25%	75%	.40

Family demographic characteristics			
Single parent	48%	52%	.46
Family size	4.05	4.30	.23
Parent post‐high school education	36%	64%	.03
Parent employed outside home	45%	55%	.94
Months of financial hardship	2.07	1.43	.05
Income‐to‐needs ratio	.87	.95	.34
Toddler gender (percentage girls)	46%	54%	.97
Toddler age in years	2.51	2.65	.001

Parent and household characteristics			
Parent with clinical depression	55%	45%	.01
Need for therapeutic services	4.12	3.57	.06
Parenting stress	2.13	2.07	.51
Household chaos	1.90	1.87	.70

*Note*: Continuous measures compared with independent two‐sample t‐test. Categorical measures are reported as percentages and compared with chi‐square test. Percentage values are reported for each characteristic (i.e., 54% of Site 1 families had a less strong working alliance, and 46% of Site 1 families had a strong working alliance).

In contrast, white families tended to have a less strong, compared to strong, working alliance, 54% versus 46%, respectively, *p* = .03. Families with more months of financial hardship in the prior year tended to have a less strong, compared to strong, working alliance, *M* = 2.07 months versus 1.43 months, respectively, *p* = .05. Parents with clinically significant symptoms of depression tended to have a less strong, compared to strong, working alliance, 55% versus 45%, respectively, *p* = .01. Likewise, families with a greater need for therapeutic services tended to have a less strong, compared to strong, working alliance, *M* rating of need = 4.12 versus 3.57, respectively, *p* = .06 (a trend).

### 3.1 Intervention effects for families with a less strong and strong working alliance

Means and standard deviations of all parent and toddler outcomes for families with a less strong working alliance are presented in Table 2, separately for families in the intervention and control conditions. Means and standard deviations of all outcomes for families with a strong working alliance are presented in Table 3.

**TABLE 2:** Descriptive statistics for families with a less strong working alliance.

	Recipe 4 success (*n* = 54)				Usual practice home visits (*n* = 56)			
	Baseline		Post intervention		Baseline		Post intervention	
	*M*	*SD*	*M*	*SD*	*M*	*SD*	*M*	*SD*
Parents’ sensitive scaffolding								
Core parenting skills	.63	.34	.79	.36	.50	.27	.58	.30
Learning support	2.97	.50	2.90	.48	2.71	.50	2.63	.55
Technical scaffolding	3.67	.63	3.64	.62	3.30	.71	3.21	.66

Food parenting practices								
Responsive strategies	.44	.11	.52	.10	.43	.13	.49	.11
Overall sensitivity	3.13	1.12	3.72	.99	3.24	1.17	3.44	1.11

Toddlers’ self‐regulation								
Delay of gratification	.57	.37	.71	.40	.57	.38	.59	.41
Task orientation	6.45	1.26	6.68	1.29	6.17	1.21	6.10	1.06
Compliance	2.31	.36	2.36	.29	2.26	.39	2.23	.32
Dysregulation	1.36	.34	1.27	.30	1.34	.27	1.32	.31

Healthy eating habits								
Eat nutritious foods	.30	.06	.32	.06	.30	.09	.32	.09
Help with meal preparation	.23	.34	.37	.35	.23	.31	.23	.32
Body mass index percentile	.95	.04	.82	.22	.94	.05	.81	.22

*Note*: Body mass index percentiles are only for toddlers with overweight/obesity at baseline.

**TABLE 3:** Descriptive statistics for families with a strong working alliance.

	Recipe 4 success (*n* = 68)				Usual practice home visits (*n* = 63)			
	Baseline		Post intervention		Baseline		Post intervention	
	*M*	*SD*	*M*	*SD*	*M*	*SD*	*M*	*SD*
Parents’ sensitive scaffolding								
Core parenting skills	.65	.32	.79	.39	.56	.30	.74	.34
Learning support	2.92	.45	2.96	.47	2.89	.45	2.87	.41
Technical scaffolding	3.47	.64	3.54	.58	3.39	.59	3.57	.56

Food parenting practices								
Responsive Strategies	.47	.12	.54	.13	.44	.12	.51	.11
Overall Sensitivity	3.48	1.09	3.68	.99	3.37	1.12	3.44	1.05

Toddlers’ self‐regulation								
Delay of gratification	.47	.40	.65	.41	.52	.40	.56	.40
Task orientation	6.27	.92	6.37	1.30	6.38	1.01	6.26	1.30
Compliance	2.31	.34	2.32	.34	2.32	.33	2.30	.29
Dysregulation	1.34	.29	1.27	.32	1.30	.30	1.27	.31

Healthy eating habits								
Eat nutritious foods	.30	.08	.33	.08	.32	.07	.31	.08
Help with meal preparation	.24	.29	.36	.35	.16	.27	.22	.33
Body mass index percentile	.93	.05	.72	.31	.94	.05	.87	.18

*Note*: Body mass index percentiles are only for toddlers with overweight/obesity at baseline.

Among families with a less strong working alliance, Recipe 4 Success, compared to usual practice home visits, resulted in notable improvements on multiple indicators of parents’ sensitive scaffolding, food parenting practices, and toddlers’ self‐regulation. As presented in Table 4, the Recipe 4 Success intervention effect on parents’ use of the four core skills hypothesized to support toddlers’ self‐regulation was *β *= .56, *p* = .003, controlling for the baseline level of those skills. An intervention effect size of this magnitude indicates that, among families with a less strong working alliance, there was an intervention‐control group difference of more than one‐half standard deviation. The Recipe 4 Success intervention effect on parents’ learning support was *β *= .32, *p* = .07 (a trend), and the intervention effect on technical scaffolding of children's learning was *β *= .46, *p* = .01. The intervention effect on parents’ use of responsive/non‐coercive food parenting practices was *β *= .32, *p* = .08 (a trend). Likewise, the Recipe 4 Success intervention effect on toddlers’ delay of gratification was *β *= .30, *p* = .09 (a trend); the intervention effect on task orientation was *β *= .40, *p* = .03; the intervention effect on toddlers’ compliance was *β *= .35, *p* = .01; and the intervention effect on the reduction of toddlers’ dysregulation, such as temper tantrums, was *β *= (− .30), *p* = .01. Finally, the intervention effect on toddlers’ involvement in meal preparation was *β *= .38, *p* = .03.

**TABLE 4:** Recipe 4 success intervention effects within parent‐home visitor working alliance subgroups.

	Less strong working alliance			Strong working alliance		
	*β*	*SE*	*p*	*β*	*SE*	*p*
Parents’ sensitive scaffolding						
Core parenting skills	.56	.19	.003	.01	.18	.96
Learning support	.32	.17	.07	.17	.16	.31
Technical scaffolding	.46	.18	.01	− .08	.18	.67

Food parenting practices						
Responsive/non‐coercive strategies	.32	.18	.08	.23	.18	.19
Overall sensitivity	.29	.19	.14	.23	.17	.19

Toddlers’ self‐regulation						
Delay of gratification	.30	.17	.09	.25	.17	.16
Task orientation	.40	.18	.03	.09	.18	.63
Compliance	.35	.14	.01	.07	.13	.60
Dysregulation	− .30	.11	.01	− .07	.11	.52

Healthy eating habits						
Eat nutritious foods	.01	.18	.94	.43	.16	.01
Involvement in meal preparation	.38	.18	.03	.27	.16	.10
Body mass index	.02	.32	.96	− .59	.26	.03

*Note*: β represent standardized intervention effect sizes, similar to Cohen's d, but controlling for the baseline level of the outcome. *SE* = standard error.

In contrast to those wide‐ranging results, among families with an already strong working alliance, Recipe 4 Success, compared to usual practice home visits, had a different pattern of intervention effects that only pertained to healthy eating habits. Among this subgroup of families, also presented in Table 4, the Recipe 4 Success intervention effect on toddlers’ eating of nutritious foods was *β *= .43, *p* = .01; the intervention effect on toddlers’ involvement in meal preparation was *β *= .27, *p* = .10 (a trend); and the intervention effect on the reduction of BMI among toddlers with overweight or obesity was *β *= (− .59), *p* = .03.

When formal tests of differences in intervention effects across families with a less strong versus strong working alliance were conducted, there was only one case, for the four core parenting skills, in which the interaction term had a probability value below . 05, and one case, for technical scaffolding, in which the probability value was from .05– .10 (a trend). Despite other substantial differences in intervention effects across families with different working alliances, all other interaction terms had a probability value above .10.

## 4 DISCUSSION

A strong working alliance between home visitors and parents is one of the key therapeutic mechanisms which support family progress in home visiting programs (Braaten et al., 2025). However, it can take time and effort to build trust in the relationship and appropriately identify family needs. When home visitors and parents have a less strong working alliance, it can be unclear how to best support families. This study helps inform our approach to working with new families or families with whom home visitors are having a harder time connecting.

In our study, about 55% of families had a strong parent‐home visitor working alliance and about 45% of families had a less strong working alliance, with clear differentiation between the two kinds of families. Understandably, we tended to observe a less strong working alliance among those families experiencing greater risks, such as financial hardship and parent depression. It is important to note, however, that the less strong working alliances were still good (with average ratings of 3.02 on a 4‐point scale). This is not surprising, given that home visitors were hired because of their special ability to connect with others. Moreover, prior to participating in this study, all families had been enrolled in Early Head Start home visiting programs which most often used the Parents as Teachers curriculum. Both Early Head Start and Parents as Teachers devote considerable resources to helping home visitors establish and maintain the strongest working alliances possible.

### 4.1 Supporting families with a less strong working alliance

The results of this study suggest Recipe 4 Success was particularly effective for families with a less strong working alliance. Among those families, Recipe 4 Success, compared to usual practice home visits, improved parents’ use of core parenting skills, learning support, technical scaffolding, and responsive/non‐coercive food parenting practices. It also improved toddler's delay of gratification, task orientation/attention, compliance, dysregulation, and involvement in meal or snack preparation.

There are multiple aspects of Recipe 4 Success that may have contributed to its effectiveness among families with a less strong working alliance. First, home visitors’ own lack of confidence and/or skill in creating and facilitating effective lessons may partly contribute to a less strong working alliance. As a result of the collective expertise of everyone involved in the community‐based participatory research collaboration (Nix, 2020), Recipe 4 Success became a comprehensive curriculum that could help bolster uncertain home visitors and serve the diverse range of families they work with.

Second, the curriculum was nearly universal in its appeal, with a focus on toddlers’ self‐regulation and enjoyable and engaging food preparation activities. Home visitors and parents did not need to identify individualized shared goals for each lesson, an important aspect of the working alliance. Moreover, home visitors did not need to create tailored lessons each week in an attempt to meet such goals. In planning meetings for Recipe 4 Success, home visitors identified what a challenge it was to come up with new lessons that were fun and rich in educational content for each family each week, even with the guidance of an evidence‐based curriculum, like Parents as Teachers. Both home visitors and parents appear to appreciate greater structure and direction during home visits (West et al., 2022). At the same time, because our lessons required less than one‐half of each home visit, home visitors could still address other pressing issues, as necessary, with each family.

Third, Recipe 4 Success incorporated the use of parent coaching strategies to promote the use of core parenting skills. The core parenting skills were borrowed from parent‐child interaction therapy, as they are highly reinforcing to toddlers and highly effective in eliciting their best behavior (McNeil &, Hembree‐Kigin, 2010). When parents actively practice using the new skills during Recipe 4 Success and receive positive feedback from their home visitors and positive responses from their children, they become more likely to use those skills in other contexts (Peterson et al., 2018). Other home visit curricula also include parent coaching. However, prior research and clinical observations suggest home visitors may be reluctant to try such directive strategies while they are still trying to develop a strong working alliance (Shanti, 2020; West et al., 2022). Worried about potential adverse consequences to their relationships with families, home visitors may opt to model rather than coach new skills, thus reducing the likelihood of parental learning. By relying on cooking as a platform to deliver our curriculum—in families’ own kitchens, no less—we inspired and gently compelled parents to be more involved in the home visits than they otherwise might have been (Braaten et al., 2025). As a result, the Recipe 4 Success curriculum afforded parents more opportunities to improve.

We hoped those aspects of Recipe 4 Success might reduce the tension home visitors may perceive between cultivating and maintaining a strong working alliance and delivering an evidence‐based curriculum (Barak et al., 2014). It may be we enhanced parents’ commitment to and engagements with future home visits by helping them realize quick gains in domains of family functioning they care about. Overall, these findings suggest that, even when home visitors have a less strong working alliance with families, reliance on a brief, highly structured parent‐coaching curriculum may be a better alternative than usual practice home visits.

### 4.2 Supporting families with whom home visitors already had a strong working alliance

Among families with whom home visitors already had a strong working alliance, Recipe 4 Success did not yield such robust intervention effects on parents’ sensitive scaffolding, food parenting practices, or toddlers’ self‐regulation. This suggests that home visits already seem to be going well for these families, in these domains of functioning. These home visitors and parents were able to collaborate to identify goals, engage in relevant activities in service of those goals, and reap the benefits of those activities without the use of an even more structured curriculum. The highly structured and detailed Recipe 4 Success curriculum appears comparable to—or only slightly better than—the lessons home visitors were already creating for each family.

The one domain in which Recipe 4 Success was especially helpful to families with a strong working alliance was healthy eating habits, which was the initial impetus for creating Recipe 4 Success. In this case, Recipe 4 Success improved the percentage of nutritious foods toddlers consumed and the likelihood they were involved in meal and snack preparation. In addition, Recipe 4 Success was associated with a reduction in BMI among toddlers with overweight or obesity. Currently, there are very few interventions that reliably change BMI among toddlers (Blake‐Lamb et al., 2016).

In part, the improvements to healthy eating habits may be due to the curriculum's food preparation activities (Braaten et al., 2025). Those activities exposed toddlers to unfamiliar foods and may have increased their motivation to eat those foods (Knai et al., 2006). Relatedly, parents may have been exposed to new recipe ideas and realized their toddlers were more receptive to trying healthy foods than parents would have predicted. In addition, parents may have learned their toddlers were capable of and interested in helping with the “grown up” cooking activity, which is associated with greater food acceptance (Knai et al., 2006).

In addition, the improvements to healthy eating habits may have occurred due to the critical combination of a strong parent‐home visitor working alliance and the curriculum. Because home visitors already had established trust in their relationships with families and because Recipe 4 Success provided the necessary background information and proper framing, home visitors may have felt more comfortable broaching the potentially sensitive topic of nutrition and healthy eating habits. Likewise, knowing home visitors had their best interest at heart and hearing how sensitively issues were addressed in the Recipe 4 Success lessons, parents may have been more receptive to such conversations and more likely to change.

### 4.3 Study strengths and limitations

In examining how working alliance may have altered the overall pattern of positive results previously observed in Recipe 4 Success, this study has several strengths. It relied on a randomized controlled clinical trial which allowed us to examine causal relations between Recipe 4 Success and improvements in family functioning. In this study, all families were already enrolled in home visits, meaning all Recipe 4 Success intervention effects depended on the foundation previously established by Early Head Start and the Parents as Teachers curriculum. However, it also meant that all intervention effects were in addition to the ones families already accrued from usual practice home visits. In this study we randomized families to study condition within home visitor caseloads. Because the same home visitors delivered lessons in both the intervention and control groups, we are able to attribute all intervention effects to the difference in curricula alone. Our sense was that home visitors did not use much of Recipe 4 Success with their control group families. Even so, any contamination across conditions would have reduced the magnitude of intervention effects we found.

Alongside its strengths, this study has notable limitations. Within‐group regression results demonstrate a compelling pattern of intervention effects among families with a less strong working alliance. In contrast, there was a distinct yet different pattern of intervention effects among families with an already strong working alliance. However, despite those obvious differences in patterns, intervention effects across subgroups were rarely statistically significant; indicating the findings in this study may be suggestive but are not definitive. Another limitation of this study is that working alliance was based on home visitors’ perceptions of their relationship with each parent prior to the start of the intervention. Results might have looked different if we had been able to include parents’ perceptions of the working alliance as well. Another limitation is that this study did not collect information about how long home visitors and parents had been working together. A less strong working alliance might have very different meanings if home visitors and parents had only been working together for a couple of months versus several years.

### 4.4 Implications for practice and conclusions

Federal funding for early childhood home visiting requires that programs use an evidence‐based curricula and model. Some of the approved curricula are narrowly focused on functioning within a very specific domain, such as parent responsiveness, and some of the curricula are designed to address a much broader range of outcomes. Moreover, some of the approved curricula are structured and standardized across families, and some are more flexible, with provisions that allow home visitors and families to set individualized goals and home visitors to identify different activities in an attempt to meet those goals. Recipe 4 Success falls near the more focused end of that continuum, as it is relatively structured and standardized across families.

This study highlights how families with a less strong working alliance may be especially likely to benefit from a more focused and structured curriculum and approach to their home visits. Such families appeared to respond well to the clear guidelines, preplanned detailed activities, and embedded coaching strategies of Recipe 4 Success. This study suggests that cultivating a strong working alliance and delivering a highly structured curriculum within home visits need not be in tension with one another, and may, in fact, support one another in better serving all families.

## CONFLICT OF INTEREST STATEMENT

The authors declare no conflicts of interest.
